# Urban street tree biodiversity and antidepressant prescriptions

**DOI:** 10.1038/s41598-020-79924-5

**Published:** 2020-12-31

**Authors:** Melissa R. Marselle, Diana E. Bowler, Jan Watzema, David Eichenberg, Toralf Kirsten, Aletta Bonn

**Affiliations:** 1 Department of Ecosystem Services, Helmholtz Centre for Environmental Research - UFZ, Permoserstraße 15, 04318 Leipzig, Germany; 2 German Centre for Integrative Biodiversity Research (iDiv) Halle-Jena-Leipzig, Puschstrasse 4, 04103 Leipzig, Germany; 3 Institute for Psychological Sciences, De Montfort University, The Gateway, Leicester, UK; 4 Institute of Biodiversity, Friedrich Schiller University Jena, Dornburger Straße 159, 07743 Jena, Germany; 5 Institute of Biology, Leipzig University, Talstraße 33, 04103 Leipzig, Germany; 6 LIFE Research Center for Civilization Diseases, Medical Faculty, Leipzig University, Philipp-Rosenthal-Str. 27, 04103 Leipzig, Germany; 7 Faculty of Computer and Biosciences, University of Applied Sciences Mittweid, Technikumplatz 17, 09648 Mittweida, Germany

**Keywords:** Biodiversity, Psychology, Epidemiology, Disease prevention, Quality of life

## Abstract

Growing urbanisation is a threat to both mental health and biodiversity. Street trees are an important biodiversity component of urban greenspace, but little is known about their effects on mental health. Here, we analysed the association of street tree density and species richness with antidepressant prescribing for 9751 inhabitants of Leipzig, Germany. We examined spatial scale effects of street trees at different distances around participant’s homes, using Euclidean buffers of 100, 300, 500, and 1000 m. Employing generalised additive models, we found a lower rate of antidepressant prescriptions for people living within 100 m of higher density of street trees—although this relationship was marginally significant (*p* = 0.057) when confounding factors were considered. Density of street trees at further spatial distances, and species richness of street trees at any distance, were not associated with antidepressant prescriptions. However, for individuals with low socio-economic status, high density of street trees at 100 m around the home significantly reduced the probability of being prescribed antidepressants. The study suggests that unintentional daily contact to nature through street trees close to the home may reduce the risk of depression, especially for individuals in deprived groups. This has important implications for urban planning and nature-based health interventions in cities.

## Introduction

Growing urbanisation is a threat to both mental ill health^1^ and biodiversity^2^. As global urban cover is projected to increase to 1.9 million km^2^ with 5.2 billion people expected to live in urban areas by 2030^2^, action is needed to reduce future risks to both people and nature. Urban nature-based solutions, such as planting trees, might be a preventative solution to tackle both mental health challenges^3^ and biodiversity loss. As such, growing the urban forest could address the nexus between sustainable and healthy cities^4^ and aid progress towards Sustainable Development Goals (SDGs) on human health and wellbeing (SDG 3), creating sustainable cities (SDG 11) and conserving terrestrial ecosystems (SDG 15)^5,6^. In order to address these SDGs–and because space in cities is scarce due to increasing urbanization–policymakers, urban planners and designers need information about which specific aspects of the urban forest influence human health.

The urban forest comprises all trees in an urban area — from individual trees to tree assemblages and forested areas on both public and private land^5,7^. In this study, we focus on a specific type of the urban forest: individual street trees. Street trees are an important component of the urban forest because: (1) they contribute to the conservation of native tree species^8^, (2) they are public amenities located throughout the urban matrix, and (3) they can be easily retrofitted into urban areas where opportunities for growing the urban forest are limited^9,10^. Street trees also provide various ecosystem services for human health and well-being, such as air quality and climate change adaptation^5,10^. To date, little is known, however, about the potential impact of street trees on mental health^10–12^.

Urban greenspace has a positive benefit on people experiencing mental ill health^11,13–15^. The focus of much of this work has been on exposure to the quantity of generic urban greenspace^14,16,17^ often measured using Normalized Difference Vegetation Index (NDVI)^17^ or the density of tree canopy^11,18^. Far less work has examined the influence of specific types^14,16,17^ or ecological quality^14,16,17,19,20^—e.g. tree species richness^16^—of urban greenspace on mental health. As mental health outcomes are influenced by the type of environment^21,22^ and its ecological quality^19^, more knowledge is needed on the types and ecological qualities of the urban greenspace that have a benefit for mental health^16,23,24^. Such evidence can inform health professionals, urban foresters, city planners, and urban designers on the required policy, planning, and management decisions necessary to ensure the urban forest has a positive impact on both public health and nature conservation^25^.

With respect to mental health, most studies on the effects of greenspace on mental health use self-reported measures^14,26^ with high variability in the number of different measures used^26^. This variability of measures used to assess mental health make comparability, and aggregating evidence on the effects of urban greenspace on mental health, difficult^26^. There is a need for more objective indices of mental health^14^. Antidepressant prescriptions provides such an objective indicator for depression prevalence^3^.

While other studies have investigated the quantity of greenspace and antidepressant prescriptions^27–29^, only one study has investigated antidepressant prescriptions in relation to street trees^30^. At an area-level, across 31 districts of London, Taylor et al.^30^ found that a higher density of street trees was significantly associated with fewer antidepressant prescriptions. To date, no study has investigated the relationship between ecological quality of street trees and antidepressant prescriptions^16^. It is therefore unknown whether ecological quality (i.e. species richness) or quantity of street trees matter.

Often, epidemiological studies on the association of greenspace exposure on mental health examine area-level effects, in which the quantity of greenspace is measured at a geographically defined area (e.g. London borough, Lower Super Output Area)^14,27^. As the previous studies examined area-level greenspace^27,28^ or street trees^30^ and antidepressant prescriptions, they are limited by the ecological fallacy—whereby relationships found at the aggregate area-level may not exist at the individual-level^16^. Individual-level data allows for a more detailed investigation of the association between street trees and antidepressants. While one study examined individual-level greenspace exposure (using NDVI) and antidepressant medication^29^, no study has yet examined individual-level exposure to ecological quality of street trees and antidepressant prescriptions.

Exposure, or amount of contact that an individual has with urban greenspace, is typically estimated through geographic metrics^3^. At an individual-level, exposure is usually based on the cumulative opportunity metric, determined as the quantity or quality of urban greenspace within a spatial buffer around the home^3,17,20^. There is no consensus on spatial distance^16,20,31^, with studies assessing the amount of greenspace within buffers ranging from 100 m^29,32^ to 3 km^33,34^. The specific hypothetical mechanism linking urban greenspace to health varies according to the size of the spatial buffer^31^. Smaller buffers may be appropriate for assessing restorative mechanisms (e.g. attention restoration, stress reduction) that may depend on views around the home^35^. Larger buffers may be more appropriate to assess physical activity as the mechanism for improved health^36,37^. Moreover, examination of urban greenspace at different spatial distances can help to understand how much nature is required to have an effect on health, answering questions about the nature intensity component of a dose^38,39^.

Another component of dose is to understand for whom urban greenspace has the strongest effect. Certain people are at greater risk for depression. In Germany, women^40^, people with low socio-economic status (SES)^40^ and unemployed people^41^ are at greater risk for depression, and are more likely to be prescribed antidepressants^42^. Previous research has investigated whether exposure to greenspace could be protective against, or moderate, health inequalities^43,44^. Specifically, the quantity of area-level greenspace has been found to moderate the negative influence of gender and residing in low SES neighbourhoods on depression^45^. It is currently unknown whether street tree quantity or quality may have a protective effect against antidepressant prescriptions for those most at risk.

In the present study we explored the influence of quantity and quality of street trees on depression, as indicated by antidepressant prescriptions. Our specific aims were: (1) to investigate the associations of street tree density and species richness in relation to antidepressant prescriptions at the individual-level; (2) to explore the importance of spatial proximity of exposure to street trees to understand the nature intensity component of dose^38^ (3) and to investigate whether street tree quantity and/or quality moderates the influence of health inequalities (gender, employment and SES) on antidepressant prescriptions. This can inform green infrastructure design and enable public health policy recommendations and planning.

## Results

**Figure 1:**
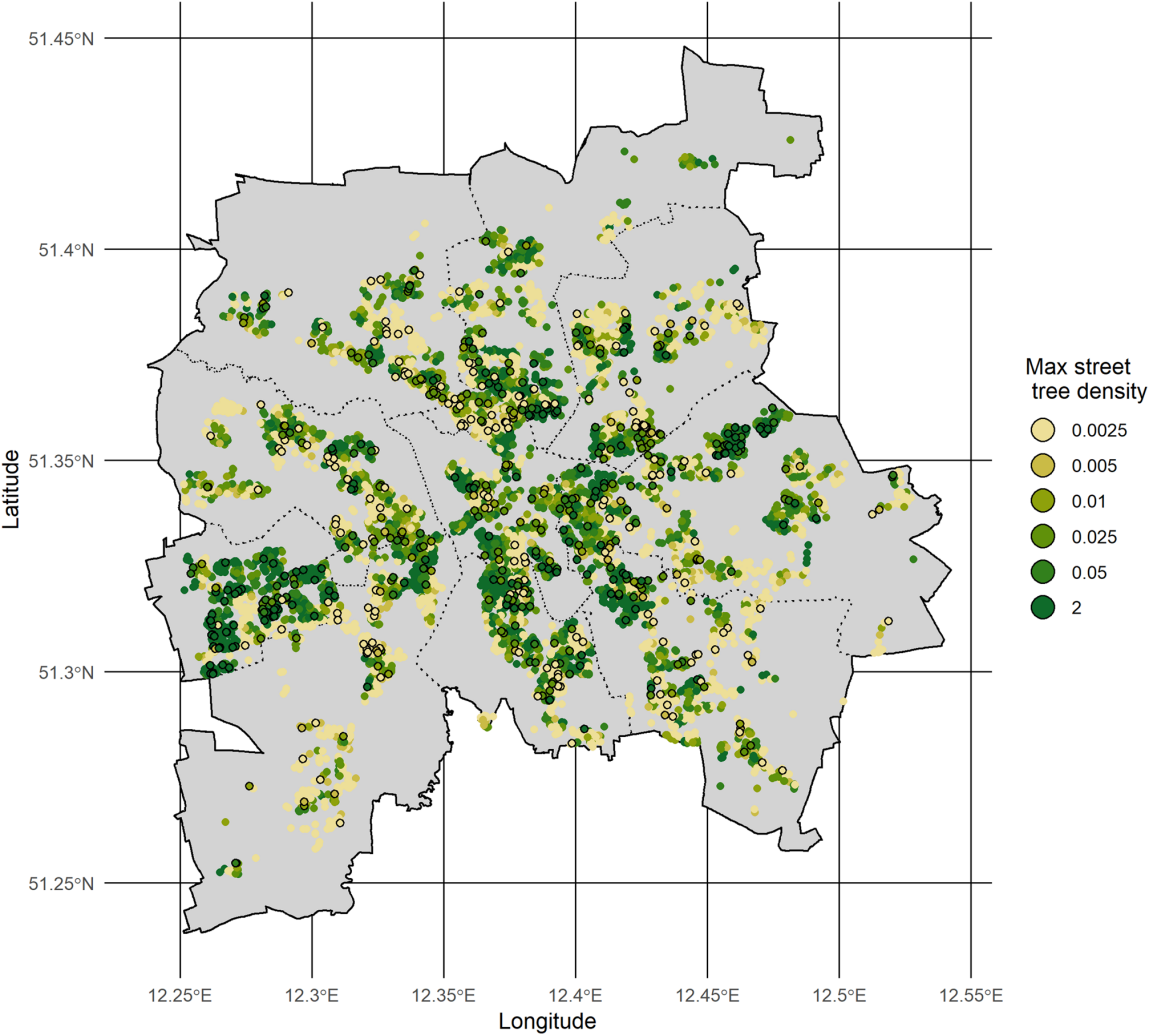
Distribution of street trees and antidepressant prescriptions amongst participants in the LIFE-Adult-Study. Circles indicate the location of participants within the city of Leipzig, Germany. Circles with a black outline represent individuals who have been prescribed antidepressants. Coloured circles shaded yellow-green reflect the density of street trees within 100 m of the home. Tree density values are number of trees per meter of road within 100 m buffer. The figure was created with ggplot2^49^ available for R (ver. 3.5.2). It is an own creation by D. Eichenberg. Polygons for the city districts were taken from City of Leipzig, Office for Statistics and Elections; Data license Germany—Attribution—Version 2.0 ^50^.

Almost 600 participants (*n* = 596, 6.1%, Supplementary Table S1) were prescribed antidepressants in the LIFE-Adult-Study. Participants, and those with prescriptions, were distributed throughout the city (see Fig. 1). With a 6% prevalence of antidepressant prescriptions, our sample is representative of all of Germany. In 2017, the average for antidepressants prescription in Germany was 56 people per every 1000 residents or 5.6% prevalence^46^.

In the city of Leipzig, street trees are planted throughout the city, while concentrated more densely in some areas than in others, with a total of 66,179 street trees, comprised of 51 genera and 131 species (Fig. 1). Supplementary Table S3 details the minimum and maximum number and their species richness of street trees around participants’ homes. Contrary to the often expected ‘luxury effect of biodiversity’^47,48^, individuals with low SES had, on average, more street trees and greater species richness of street trees around their homes, compared with the other SES groups at both 100 m and 1000 m (Supplementary Table S4).

**Figure 2:**
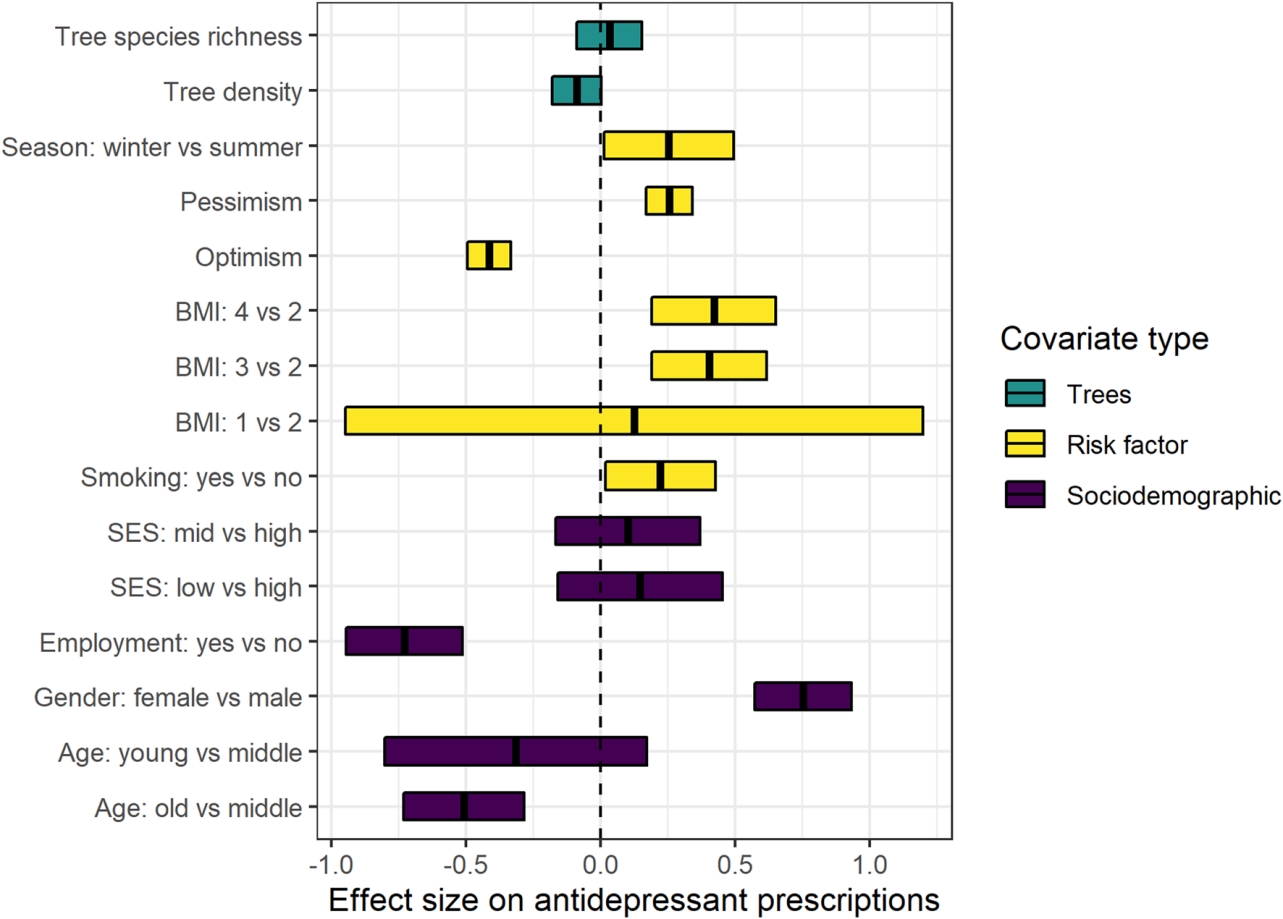
Effect size of covariates and street tree density and richness at 100 m around the home on antidepressant prescriptions. Shown are the regression coefficients (change in log OR) and 95% confidence intervals. Regression coefficients for continuous variables (pessimism, optimism, tree richness and density) were scaled to units of standard deviation; for the categorical variables, the effect sizes represent differences between levels. The dashed line is the line of no effect. BMI (Body Mass Index): 1 = underweight, 2 = normal weight, 3 = overweight, 4 = obese. Age: young = 18–39 years; middle = 40–64 years; old = 65–79 years. Imputed cases dataset (*n* = 9571).

**Figure 3:**
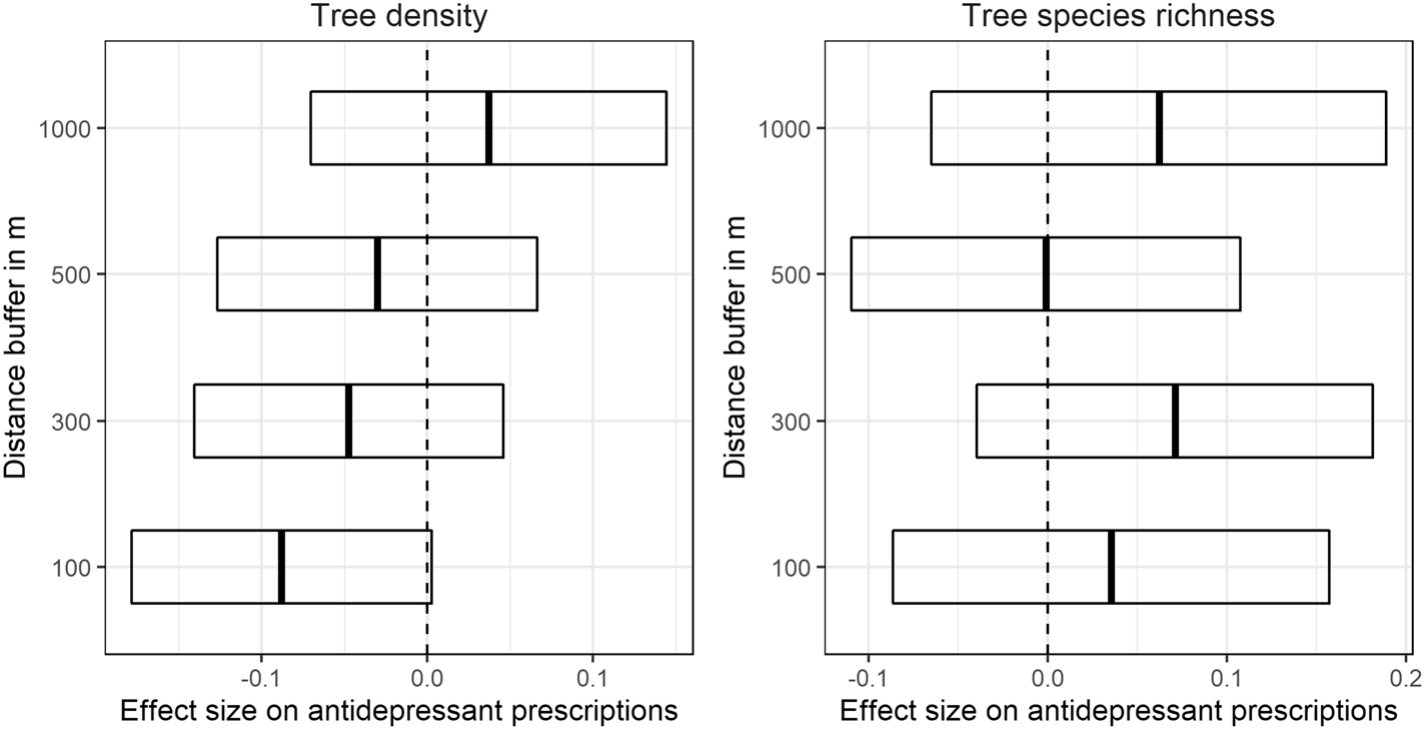
Effect size of street tree density and species richness at different spatial distances around the participants’ home (buffer widths) on antidepressant prescriptions. Street tree density and richness were standardized to units of standard deviation prior to analysis. Imputed dataset (*n* = 9571).

Greater risk of antidepressant prescriptions was associated with being female, overweight or obese, a smoker, pessimistic, and the seasons of winter and spring (Fig. 2; Supplementary Table S5). By contrast, reduced risk of antidepressant prescriptions was associated with being young (18–39) or old (age 65 +), employed, and optimistic (Fig. 2). Accounting for covariates, people living in homes with greater density of street trees within 100 m were less likely to be prescribed antidepressants, although this relationship was marginally significant (log OR =  −0.09; SE = 0.05; 95% CI − 0.18 to 0.00; *p* = 0.057; Fig. 2). No significant association between density of street trees and antidepressant prescriptions were found at greater distances (Fig. 3). Species richness was not significantly associated with antidepressant prescriptions at any distance (Fig. 3).

**Figure 4:**
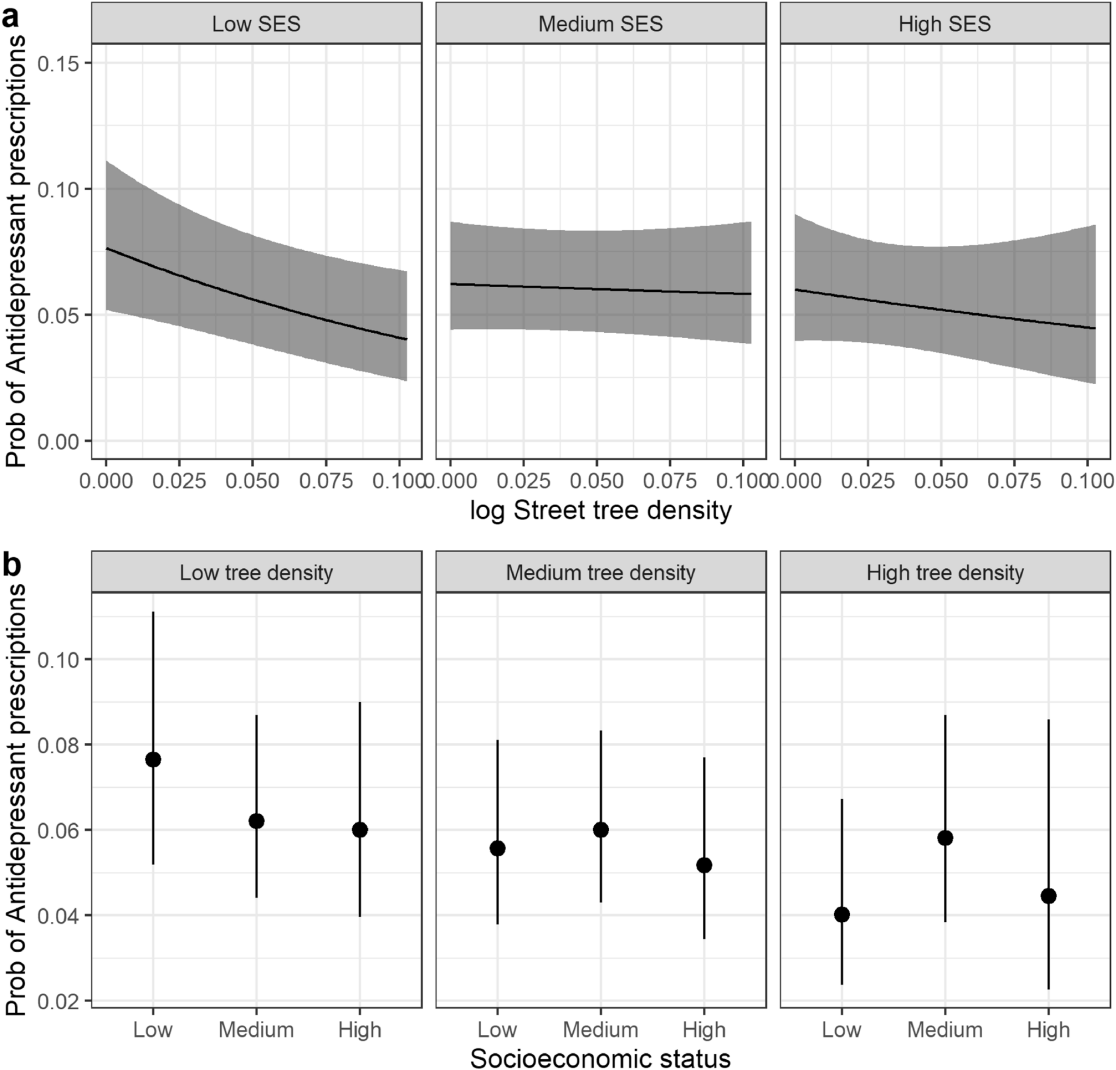
(**a**) Probability of antidepressant prescriptions as a function of street tree density 100 m around the home and individual socio-economic status (SES). The black line is the mean and the shaded area are the 95% confidence intervals. The regression is statistically significant ( *p*< 0.01) for low SES but not for medium or high SES. (**b**) Probability of antidepressant prescriptions as a function of low (0), medium (average) and high (max) street tree density 100 m around the home stratified by SES. The black dot is the mean and the black line is the 95% confidence interval.

Subsequently, in the moderator analyses, we concentrated on street tree density 100 m around the home. Overall, the effect of street tree density did not significantly moderate the effect of SES (interaction test: χ^2^ = 3.95, df = 2, *p* = 0.14). This was due to the large uncertainty (standard error) of street trees for the high SES group (Supplementary Fig. S1). However, in the stratified analyses for each SES group, there was a significant negative effect of street tree density on antidepressants for the low SES group only (log OR =  −0.21, SE = 0.08, *p* = 0.01). Greater density of street trees at 100 m reduced the probability of someone with low SES being prescribed antidepressants (Fig. 4a). However, for the medium and high SES groups, the effect of street tree density at 100 m from the home did not significantly change the probability of being prescribed antidepressants (medium SES: log OR =  −0.02, SE = 0.06, *p* = 0.72; high SES: log OR =  −0.10, SE = 0.12, *p* = 0.44). (Fig. 4a). The net result of these differences is that under low street tree density, individuals with low SES tended to have higher probabilities of antidepressant prescriptions (Fig. 4b), but under high street tree density, individuals with low SES tended to have similar probability of being prescribed antidepressants as individuals with high SES. In other words, differences in the probability of being prescribed antidepressants between those with lowest and highest SES fell as street tree density improved. For gender and employment status, there was no significant interaction and estimated effect sizes were similar for each group in the stratified analyses (i.e., for males and females, and employed and unemployed) (Supplementary Table S6).

## Discussion

We present a novel analysis of the association between street tree density and biodiversity on mental health. Our analysis found some evidence that higher density of street trees 100 m around the home was associated with fewer antidepressant prescriptions, albeit this was marginally significant after controlling for covariates. In stratified analyses, we found a significant association of higher street tree density on lower antidepressant prescriptions for individuals with low SES. We found no significant effect of street tree species richness on antidepressant prescriptions. It is important to note that we found no evidence of a luxury effect of biodiversity^47,48^ in our sample, as median street tree density 100 m was significantly higher for individuals with low SES.

Our marginally significant, negative association between street tree density at 100 m and antidepressant prescriptions is consistent with studies that found negative associations between quantity of area-level greenspace and antidepressant prescriptions^27,28,30^. Although this relationship was nonsignificant in the Gidlow et al.^27^ study. At the individual-level, similar beneficial effects were also found for nearby urban greenspace and self-reported intake of antidepressants^29^, and depression^51^.

While low socio-economic status is related to higher prevalence of depression^40,41^, our study found street tree density 100 m around the home significantly lowered the risk of being prescribed antidepressants for people with low SES. Indeed, under high density of street trees at 100 m, individuals with low SES had a similar probability of being prescribed antidepressants as individuals with high SES. Similar results regarding the protective benefit of urban greenspace for socio-economically deprived populations have previously been found^33,43–45,52,53^. Some pathways through which low SES might lead to worse mental health are possibly modified by exposure to nearby nature. These mechanisms are physical activity and psychological restoration (e.g. attention restoration, stress reduction)^36,37^. The close spatial proximity to nearby street trees at 100 m around the home implies the underlying mechanism linking street trees to depression could be psychological restoration^31,36,37^, rather than physical activity. Previous research has found that people whose homes had views of high amounts of diverse vegetation had lower stress^54^ and greater restoration^55^. Adults with low SES living in social housing who had a view of nearby trees from their homes reported greater attentional functioning and life management effectiveness^56^. As stress and poor attentional functioning are risk factors for depression^57^, and these risk factors are experienced more in individuals with low SES^58,59^, reducing these risk factors might have contributed to the reduced prevalence of antidepressants for those with low SES who live in areas with high density of street trees near the home.

Overall, our results suggest that the quantity of street trees around the home may be more important for preventing depression than the ecological quality of street trees, i.e. species richness. This finding is supported by other studies that found abundance of a taxonomic group—but not its species richness—affects mental health and wellbeing^60–62^. Shanahan et al.^38^ found no association between depression and vegetation complexity, a measure related to plant diversity. An experimental study in China found walks along roads, each with a different species of street tree, resulted in better mental health compared to walks in a road without street trees^12^, suggesting the mere presence of trees on streets, but not their species affiliation, is important. Given that most people cannot identify different plant species in general^60^, benefits of street trees may rather be provided through people experiencing tree abundance^61^. Planting and maintaining street trees thereby provides a proactive public health measure that also meets conservation goals.

To address questions about the dose^39^, i.e. ‘how much’ street trees are required to have an effect on antidepressant prescriptions, we examined the effect of different spatial distances of street tree density and species richness around people’s homes. The general assumption is that, for cumulative opportunity metrics, more greenspace offers more opportunity for nature contact, and thus more opportunities for receiving its health benefits^17^. In our study, it was the at the closest spatial proximity to the home in which tree density had a marginally significant effect on reducing the likelihood of antidepressant prescriptions, suggesting that daily contact with nearby nature may be important for mental health. People experience street trees near the home as part of their daily lives, e.g. viewed through residential windows or when travelling to/from the home^9,54,63,64^. Indeed, the most common way people experience nature is through a window^65^. This everyday contact with nearby nature—either through a window view at the home^35,54,66^ or on the street^18,51^—has been shown to be beneficial for mental health and wellbeing. During the COVID-19 pandemic when people were encouraged to stay-at-home, those who had views of trees and greenspace from the home reported lower rates of depression and anxiety^67^. These studies suggest that ‘unintentional’^17^ contact with nearby nature in daily life is important for mental health. While planning guidance for urban greenspace is mostly based on intentional, purposeful visits for recreation^17^, we suggest that such ‘unintentional’ everyday contact may reach more people and that such easily accessible urban greenspace can contribute to public health.

While this study deepens our understanding of the influence of a specific type of the urban forest—individual street trees—and its quantity and ecological quality on mental health, it does have several limitations. First, due to the cross-sectional study design, we are unable to draw firm conclusions about causality of the relationships. The results may be influenced by selective migration in which healthy people choose to live in neighbourhoods with more street trees. However, as mentioned above, in our sample people with low SES lived in areas with more trees, i.e. there was no biodiversity luxury effect. Second, while antidepressant prescriptions are an objective measure of depression, they naturally only serve as a proxy for the prevalence of clinical depression. Not all individuals with depression may receive a prescription for antidepressants. Whether or not an individual with depression is prescribed antidepressants depends on other factors, such as how depression is diagnosed and the medical accessibility or availability of treatment options^3,30,46^. Third, other measures of the urban forest (e.g. tree canopy, land cover) can also influence mental health^11^. In contrast to previous studies^18,30,51^, we had specific data on street tree distributions around individuals' homes. Unfortunately, similar individual level data for non-street trees, as we had for street trees, was not available. Hence, we were unable to control for the effects of other types of greenspace, like non-street trees, which may have also affected antidepressant prescription rates. Further work might compare the relative importance of street trees compared with trees in other contexts, such as park trees. Finally, while large, old trees are recognized for their ecological value^68,69^, as well as potential for psychological restoration^70^, we were unable to analyse the influence of tree size, as these data were not available in the street tree dataset. Future studies may wish to investigate how functional characteristics of individual street trees, such as tree size^70^ or height^65^, may be related to lower depression prevalence.

## Conclusions

We live in an increasingly urban world^2^. The future of biodiversity conservation and people’s health depends on urban landscapes and respective urban planning decisions. Our finding suggests that street trees—as small-scale, publicly accessible urban greenspace—could contribute to an “equigenic environment”^44^, i.e. nature-based solutions that can help close the gap in health inequalities between individuals with low and high SES. Incorporating unintentional nature experience into everyday life around the home could be important for mental health. As such, street trees should be planted equally throughout a city to ensure those who are socially disadvantaged have equal access to nearby nature, thereby safeguarding urban health equity and preventing green gentrification^71^. As employees with low SES are more likely to be prescribed antidepressants^42^, ecosystem service accounting models can quantify the financial saving to employers and the public health system following such street tree planting projects^3^. Street trees planted equitably in residential areas may provide nature-based solutions for cities to achieve nexus of SDG targets^4–6^ relating to health and wellbeing (SDG 3.4 “promote mental health and well-being”), sustainable cities (SDG 11.7 “provide universal access to safe, inclusive and accessible, green and public spaces”), reducing inequality (SDG 10.3 “ensure equal opportunity and reduce inequalities”) and conserving terrestrial ecosystems (SDG 15.9 “integrate ecosystem and biodiversity values into…local planning”)^72^. We propose that retrofitting greenspace, namely street trees, in urban areas may promote mental health, reduce social inequalities and contribute to multiple Sustainable Development Goals^4,5^.

## Methods

We tested the association of street tree density and species richness on antidepressant prescriptions using health data from 9751 adults aged 18–79 and publicly available street tree data.

### Study population

Individual-level health data were collected from the first wave (2011–2014) of the longitudinal epidemiological study LIFE-Adult-Study of Leipzig, Germany^73^. Written informed consent was obtained from all participants before they were included and examined in the LIFE-Adult-Study^73^. This study was approved by the ethics committee of the Medical Faculty of the University of Leipzig. Data was been provided by the LIFE Research Center (analysis project proposal No. 389) and analysed in accordance with the data protection regulations of the University.

Participants in the LIFE-Adult-Study comprised a random sample of 10,000 adults aged 18–79 who lived in Leipzig, Germany^73^. Geographic coordinates were available for 9764 participants. For anonymity, geographic coordinates were randomly modified (or fuzzed) within a 30 m Euclidean buffer around the original location of participant’s home addresses. Thirteen participants were excluded because of overlapping Euclidean buffers due to the same geographical coordinates. The final sample size was 9751.

### Antidepressant prescription data

Data on antidepressant medication taken in the previous 7 days were gathered during interviews in the LIFE-Adult-Study^73^. These data were collected in all months of the year starting in April 2011 and ending in November 2014. During the interviews, all medications taken in the previous 7 days were identified by barcodes, and coded using the Anatomical Therapeutic Chemical (ATC) classification system^73^. Antidepressants are defined here as medications that start with ATC code N06A^74^. For details on the types of antidepressant included, see the Supplementary Information.

### Street tree data

Data on the location and species affiliation of public street trees throughout the city were obtained from the City of Leipzig^75^. The tree cadastre was published in February 2015 (https://opendata.leipzig.de/dataset/strassenbaumkataster). The median planting year of the street trees was 1996. For details on the data processing, see the Supplementary Information. Assessment of street tree abundance and species richness was based on each participants’ home address. To address the relevance of spatial proximity of exposure, we created Euclidean buffers at 100, 300, 500, and 1000 m around the home address of each study participant, based on previous literature^29,31^. Street tree quantity (measured as abundance, i.e. total number of all trees, irrespective of their species affiliation) and quality (measured as species richness, i.e. total number of tree species) was then calculated within each buffer. As street trees are dependent on the presence of public streets, street tree abundance was converted into street tree density by dividing by the length of streets within each buffer (abundance/road length in meters)^30^ in order to account for this dependency. The total length of streets within each buffer was calculated in QGIS using road data from OpenStreetMap (see Supplementary Information for details).

### Individual covariates and confounders

Variables previously shown to be correlated with depression were included to adjust for the other influences on depression. Socio-demographic covariates related to depression prevalence included were age^40^, gender^40^, and marital status^76^. Socio-demographic confounders included were employment status^41^, net income^40^, and socioeconomic status (SES)^40,77^. Behavioural factors, such as alcohol consumption^78^ and smoking behaviour^79^ and body mass index (BMI)^80^, were included as all are related to depression prevalence. The season of the year (winter/spring/summer/autumn), when the medication data were collected in the LIFE-Adult-Study, was included as seasonal variation may be related to depression^81^. Dispositional optimism and pessimism^82^ were included as a covariate because it is a predictor of depression^82,83^. Data for all covariates and confounders were at an individual level and were obtained from the LIFE-Adult-Study. All variables are described in detail in Supplementary Table S2.

### Statistical analyses

Due to some missing data in covariates and confounders, a random forest model (using the *missForest* R package) was used to impute missing values. *missForest* is a nonparametric imputation method that includes any nonlinear or complex associations among variables. The analysis was run with both imputed and complete case (original) datasets and almost identical results were found. We report analyses with the imputed dataset in the main text. See Supplementary Information for analyses with the complete cases (original) dataset.

The association of street tree density and species richness on antidepressant prescriptions was tested using generalised additive models (library mgcv of R, version 1.8-27), assuming a binomial error distribution for antidepressants prescribing (prescribed or not-prescribed). Effect sizes represent the change in log odds ratio (OR) of antidepressant prescribing per unit change in the covariate or independent variable. Duchon spline terms for latitude and longitude were included to model effects of the spatial structure of participant’s residential addresses. We first tested the potential covariates in a multiple regression model. Marital status, net income, SES, and alcohol use were nonsignificant predictors of antidepressant prescriptions. All variables, except SES, were removed from subsequent analyses. SES was kept in these models, regardless of significance in the models, due to its importance as a potential confounder based on previous results in the literature. We then tested the effect of street trees (richness or density) in a further model that included all these significant covariates. Since species richness tends to increase with the amount of trees^84^, street tree abundance was included as a covariate in the models testing the effect of street tree species richness on antidepressant prescribing. Tree density was log-transformed since it was strongly right-skewed. Further, due to some still extreme high values, the top 5% values were capped to the value of the 5% upper quantile to avoid our results being driven by outliers; however, similar results were found when street tree density was not capped. Analyses of the effects of street tree density and species richness were repeated at the four spatial scales (100 m, 300 m, 500 m and 1000 m buffers).

To test whether the association between socio-demographics and antidepressants varied by exposure to street trees, interaction terms were included between any significant street tree variable and (1) gender, (2) SES, and (3) employment status. Two types of analyses were run. First, we formally tested the interaction coefficient (moderator × street trees). Second, we preformed stratified analyses of the effect of street trees on antidepressant prescriptions for each level of the moderator variable (e.g., estimated the effect of street trees for low, medium and high SES groups separately). Continuous covariates were centred to their means and scaled to units of standard deviation prior to this analysis. We examined variance inflation factors for the models to check there was no evidence of multi-collinearity (none found). R version 3.5.0 was used for all analyses. All statistical tests were two-sided.

To see whether there was evidence of a ‘luxury effect of biodiversity’^47,48^, Kruskal–Wallis Test were used to examine differences in median street tree density and species richness at 100 m and 1000 m around the home according to participants’ SES.

## Supplementary Information

Supplementary Information.

## Data Availability

The health data that support the findings from this study are available from the Leipzig Research Centre for Civilization Diseases (LIFE) Research Centre (http://life.uni-leipzig.de/en/life_health_study.html), but restrictions apply to the availability of these data, which were used under license for the current study, and so are not publicly available. Data are however available following permission from the LIFE Research Centre and the ethics committee of the Medical Faculty of the University of Leipzig. The street tree data that support the findings of this study are freely available from the City of Leipzig (https://opendata.leipzig.de/dataset/strassenbaumkataster). Data on streets in Leipzig, used to determine street tree density in this study, is available from OpenStreetMap (http://download.geofabrik.de/europe/germany/sachsen.html).
