# Supplementary material for: Urban street tree biodiversity and antidepressant prescriptions
Source: Sci Rep. 2020 Dec 31;10:22445. doi: 10.1038/s41598-020-79924-5 (PMC7775428; doi:10.1038/s41598-020-79924-5)
Supplement: Supplementary file 1 — Supplementary Information. [file 41598_2020_79924_MOESM1_ESM.docx]

# Urban street tree biodiversity and antidepressant prescriptions

# Supplementary Information

Melissa R. Marselle^1,2^*, Diana E. Bowler^4,1,2^, Jan Watzema^1,2^, David Eichenberg^5,1,2^, Toralf Kirsten^6,7^ & Aletta Bonn^1,4,2^

^1.^ Helmholtz Centre for Environmental Research - UFZ, Department of Ecosystem Services, Permoserstraße 15, 04318 Leipzig, Germany

^2.^ German Centre for Integrative Biodiversity Research (iDiv) Halle-Jena-Leipzig, Deutscher Platz 5e, 04103 Leipzig, Germany

^3.^ Institute for Psychological Sciences, De Montfort University, The Gateway, Leicester, United Kingdom

^4.^ Institute of Biodiversity, Friedrich Schiller University Jena, Dornburger Straße 159, 07743 Jena, Germany

^5.^ Leipzig University, Institute of Biology, Talstraße 33, 04103 Leipzig, Germany

^6.^ LIFE Research Center for Civilization Diseases, Leipzig University, Medical Faculty, Philipp-Rosenthal-Str. 27, 04103 Leipzig, Germany

^7.^ Faculty of Computer and Biosciences, University of Applied Sciences Mittweid, Technikumplatz 17, 09648 Mittweida, Germany

1. **Supplementary Methods**
2. **Supplementary Tables**
3. **Supplementary Figures**
4. **Complete cases (original data) analyses**
5. **Supplementary References**

**1. Supplementary Methods**

**1.1 Additional data processing**

***1.1.1. Processing of street tree data***

Species name in the dataset was checked for accuracy using the Taxonomic Name Resolution Service^1,2^. Any species names identified as ‘unknowns’, ‘synonyms’ and ‘no opinions’ by the TNRS were resolved by an ecologist (DE) (e.g. *Platanus x acerifolia* resolved to *Platanus acerifolia*; *Salix matsudana* resolved to *Salix babylonica*)*.* Simple varieties of a distinct species were replaced with the distinct species names (e.g. *Robinia 'Casque'* was changed to *Robinia margaretta*). Complex varieties that are hybrid species, bred from two or more species (e.g. *Ulmus 'Dodoens'*) were treated as own species in order to account for the ‘aesthetic effect’ of these trees. From a biodiversity /ecosystem functioning perspective, the conservative action would be to ignore the specific variety (e.g. *Ulmus ‘Dodoens’* and *Ulmus ’Lovel’* would both be classified as ‘*Ulmus* variety’). However, from a psychological perspective, retaining the variety information might well be important, though, and the variety in complex varieties was retained. These retained complex varieties were identified by their parental species (e.g. *Ulmus 'Dodoens'* became *Ulmus exoniensis x U. wallachiana*). Ambiguous species names (e.g. *Platanus x acerifolia; Tilia x vulgaris 'Pallida'*) were included in the dataset.

***1.1.2 Antidepressants included in the study***

Data on antidepressant medication taken in the previous 7 days were gathered during interviews in the LIFE-Adult-Study^3^. These data were collected in all months of the year starting in April 2011 and ending in November 2014. All medications were identified by barcodes, and coded using the Anatomical Therapeutic Chemical (ATC) classification system^3^. Antidepressants are defined here as medications that start with ATC code N06A^4^.

ATC code N06A comprises mediations “used in the treatment of endogenous and exogenous depressions. The group is subdivided mainly according to mode of action. The various antidepressants have different modes of action, and the classification will not reflect the exact mode of action of the various antidepressants”^4^. The following groups of antidepressant medications starting with ATC code N06A^4^ were included in this study:

N06AA **Non-selective monoamine reuptake inhibitors** (e.g. ATC code: N06AA01 Name: desipramine)
N06AB **Selective serotonin reuptake inhibitors** (e.g. ATC coode: N06AB02; Name: zimeldine)
N06AF **Monoamine oxidase inhibitors, non-selective** (e.g. ATC code: N06AF01; Name: isocarboxazid)
N06AG **Monoamine oxidase A inhibitors** (e.g. ATC code N06AG02; Name: moclobemide)
N06AX **Other antidepressants** (e.g. ATC code: N06AX01; Name: oxitriptan).

***1.1.3 Open Street Map***

Street length was calculated using road data from OpenStreetMap (OSM)^5^. Open Street Map (OSM) is a collaborative, open-source (wiki) project to create a free and editable map of the world. This means that any registered user can participate in the project by adding or changing data.

OSM has procedures in quality-assurance procedures place for ensuring accuracy of its data from users (http://osmlab.github.io/osm-data-quality/). The accuracy and validity of OSM has been compared against other non-user generated online maps (i.e Google Maps, Bing Maps)^6^, commercial maps (i.e. TomTom)^7,8^ and land survey data ^7,9^. These studies found that OSM is similar accuracy to online and commercial maps and land survey data*.*

***1.1.3 List of OSM road classes included in road length calculation***

The following OSM road classes were included in the calculation of road length within each buffer:

- Roads and Link Roads:
  - Primary
  - Primary link
  - Residential
  - Secondary
  - Secondary link
  - Tertiary
  - Tertiary link
  - Unclassified
- Special road types
  - Living streets
  - Service road

Excluded were the following road classes from the calculation of road length within each buffer:

- Roads and Link Roads:
  - Motorway
  - Motorway link
  - Trunk
  - Trunk link
- Special road types:
  - Pedestrian
  - Tracks
  - Raceway
- Paths
  - Footway
  - Bridleway
  - Steps
  - Path
  - Cycleway

The rationale for selection of road classes was to capture the streets and roads that have public street trees; for this reason, motorways, truck roads and raceways are excluded. We also did not wish to double count road lengths by including road classes that may be present next to other road classes (e.g. street trees planted alongside a road and cycleway), thus resulting in double counting of road length. For this reason, cycleways were excluded. We did not want to ‘dilute’ street tree density by including additional lengths of road classes that appear throughout the city, and which do not exclusively have public street trees (e.g. footways and paths are found in parks in which park trees but not street trees are planted). For this reason, pedestrian, bridleways, footways, paths, steps and tracks were excluded.

Definitions of each road class can be found in the OSM wiki https://wiki.openstreetmap.org/wiki/Key:highway.

**2. Supplementary Tables**

**Supplementary Table S1.** Characteristics of the imputed data sample (*n* = 9751) and those who were and were not prescribed antidepressants by socio-demographic, lifestyle and personality factors.

|  | Participants prescribed  antidepressants  (*n* = 596) | | Participants not prescribed antidepressants  (*n* = 9155) | | Total sample  (*n*=9751) |  |
| --- | --- | --- | --- | --- | --- | --- |
|  | *n* | % | *n* | % | *n* | % |
| *Age* |  |  |  |  |  |  |
| Mean (SD) | 58.85 (10.96) | | 57.40 (12.53) | | 57.49 (12.44) | |
| *Age group* |  |  |  |  |  |  |
| Young (18-39 years) | 12 | 2.0 | 475 | 5.2 | 487 | 5.0 |
| Middle (40-64 years) | 392 | 65.8 | 5701 | 62.3 | 6093 | 62.5 |
| Old age (65-79 years) | 192 | 32.2 | 2979 | 32.5 | 3171 | 32.5 |
| *Gender* |  |  |  |  |  |  |
| Male | 193 | 32.4 | 4467 | 48.8 | 4660 | 47.8 |
| Female | 403 | 67.6 | 4688 | 51.2 | 5091 | 52.2 |
| *Marital Status* |  |  |  |  |  |  |
| Married (living together or separately) | 355 | 59.6 | 5745 | 62.8 | 6100 | 62.6 |
| Single | 86 | 14.4 | 1637 | 17.9 | 1723 | 17.7 |
| Divorced | 102 | 17.1 | 1229 | 13.4 | 1331 | 13.6 |
| Widowed | 53 | 8.9 | 544 | 5.9 | 597 | 6.1 |
| *Employment status* |  |  |  |  |  |  |
| Unemployed, or working less than 15 hrs/week | 376 | 63.1 | 4248 | 46.4 | 4624 | 47.4 |
| Employed, or working 15+ hrs/week | 220 | 36.9 | 4907 | 53.6 | 5127 | 52.6 |
| *Net Income (*€*)* |  |  |  |  |  |  |
| <1100 | 207 | 34.7 | 1980 | 21.6 | 2187 | 22.4 |
| 1100-2000 | 304 | 50.1 | 5121 | 55.9 | 5425 | 55.6 |
| >2000 | 85 | 14.3 | 2054 | 22.4 | 2139 | 21.9 |
| *Socio-economic status^1^* |  |  |  |  |  |  |
| Low | 187 | 31.4 | 1770 | 19.3 | 1957 | 20.1 |
| Medium | 337 | 56.5 | 5529 | 60.4 | 5866 | 60.2 |
| High | 72 | 12.1 | 1856 | 20.3 | 1928 | 19.8 |
| *Body Mass Index* |  |  |  |  |  |  |
| Underweight | 3 | 0.5 | 54 | 0.6 | 57 | 0.6 |
| Normal weight | 147 | 24.7 | 3188 | 34.8 | 3335 | 34.4 |
| Overweight | 255 | 42.8 | 3679 | 40.2 | 3934 | 40.3 |
| Obese | 191 | 32.0 | 2234 | 24.4 | 2425 | 24.9 |
| *Smoking status* |  |  |  |  |  |  |
| Current non-smoker | 440 | 73.8 | 7223 | 78.9 | 7663 | 78.6 |
| Current smoker | 156 | 26.2 | 1932 | 21.1 | 2088 | 21.4 |
| *Alcohol consumption (g/day)* |  |  |  |  |  |  |
| <20g/day | 522 | 87.6 | 7411 | 81.0 | 7933 | 81.4 |
| ≥20g/day | 74 | 12.4 | 1744 | 19.0 | 1818 | 18.6 |
| *Season* |  |  |  |  |  |  |
| Winter | 151 | 25.3 | 1982 | 21.6 | 2133 | 21.9 |
| Spring | 181 | 30.4 | 2308 | 25.2 | 2489 | 25.5 |
| Summer | 142 | 23.8 | 2497 | 27.3 | 2639 | 27.1 |
| Autumn | 122 | 20.5 | 2368 | 25.9 | 2490 | 25.5 |
| Optimism^2^ |  |  |  |  |  |  |
| Mean (SD) | 7.51 (2.62) | | 8.82 (2.38) | | 8.74 (2.42) | |
| Pessimism^2^ |  |  |  |  |  |  |
| Mean (SD) | 5.46 (2.49) | | 4.46 (2.31) | | 4.52 (2.33) | |

*Note*. Imputed dataset (*n* = 9751). ^1^ = Index of social class^10^ derived from information on education, income and current (job) position. ^2^= Continuous variable (range 0 -12).

**Supplementary Table S2**. Descriptions of the covariates and confounders examined in the statistical analysis.

| **Variable name** | **Description** |
| --- | --- |
| Age | Respondent’s age in years, grouped into three age categories: Young (18-39 years); Middle (40-64 years); and Old (65-79 years) age. |
| Gender | Participant’s gender. For analysis purposes, male = 1, female = 2, with male as the reference group. |
| Marital Status | Respondent’s marital status: married = 1; single = 2; divorced = 3; widowed = 4. For analysis purposes, married was used as the reference group. |
| Employment status | Respondent’s employment status, grouped into two categories^11^: Unemployed (defined as working less than 15 hours per week, includes unemployed individuals as well as retired adults, students, housewife, houseman, those on parental leave and in military service) = 0; Employed (working more than 15+ hours per week) = 1. |
| Net Income (€) | Respondent’s monthly net income, categorized^12,13^ as <€1100; €1100-€2000; and >€2000. For analysis purposes the variable was treated as a numeric ordinal. |
| Socio-economic status (SES) | Respondent’s personal socio-economic status, is a points based system comprising the individual’s academic and professional qualifications, the highest occupational status of the household, and household net income^10^. SES was grouped into three categories^10,13^: low = 1, medium =2 and high = 3. For analysis purposes, High SES was used as the reference group. |
| Body Mass Index (BMI) | Respondent’s BMI, grouped into four categories^14,15^: underweight = 1, normal weight = 2; overweight = 3; obese = 4. For analysis purposes, normal weight was used as the reference group. |
| Smoking status | Respondent’s current smoking status, grouped into two categories^12,13^: 0 = current non-smoker (includes former smoker); 1 = current smoker. For analysis purposes, current non-smoker is reference group. |
| Alcohol consumption (g/day) | Respondent’s alcohol intake of alcoholic beverages (beer with normal or higher alcoholic strength, wine, sparkling wine or fruit wine, sprits), grams per day, grouped into two categories^12,13^: <20g/day = 0; ≥20g/day = 1. |
| Season of medication data was collected | Season (based on date) when participant’s medication data was collected, grouped into four categories: winter, spring, summer, and autumn. For analysis purposes, summer was used as the reference group. |
| Optimism | Respondent’s level of dispositional optimism, measured with the German Life Orientation Test (LOT-R)^16,17^. Participants answer three items on a five point scale (0 = strongly disagree, 4 = strongly agree)^18^, resulting in a minimum score of 0 and a maximum score is 12. Higher scores indicate greater optimism. |
| Pessimism | Respondent’s level of dispositional pessimism, measured with the German LOT-R^16,17^. Participants answer three items on a five point scale (0 = strongly disagree, 4 = strongly agree)^18^, resulting in a minimum score of 0 and a maximum score is 12. Higher scores indicate greater pessimism. |

**Supplementary Table S3.** Minimum (min) and maximum (max) species richness and abundance of street trees around the study participants’ homes, depending on buffer size.

|  | Buffer sizes | | | |
| --- | --- | --- | --- | --- |
|  | 100m | 300m | 500m | 1000m |
| Street tree species richness (min-max) | 0 - 17 | 1 - 33 | 1 - 49 | 1 - 58 |
| Street tree abundance  (min-max) | 0 - 123 | 0 - 960 | 0 - 2184 | 9 - 5244 |

**Supplementary Table S4**. Median of street tree density and species richness at buffer sizes 100m and 1000m around the home for individuals with low, medium and high socio-economic status (SES).

| Street tree type and spatial scale | SES | | |  |  |  |
| --- | --- | --- | --- | --- | --- | --- |
|  | Low | Medium | High | Kruskal-Wallis chi-squared | df | *p*-value |
| Buffer size: 100m | | | |  |  |  |
| Median (range) street tree density | 0.022  (0.002-0.056) | 0.018  (0.000-0.048) | 0.015  (0.000-0.047) | 24.229 | 2 | <.001 |
| Median (range) street tree species richness | 2 (1-4) | 2 (0-4) | 2 (0-3) | 51.126 | 2 | <.001 |
| Buffer size: 1000m | | | |  |  |  |
| Median (range) street tree density | 0.025  (0.017-0.036) | 0.024  (0.017-0.035) | 0.025  (0.018-0.036) | 12.562 | 2 | 0.002 |
| Median (range) street tree species richness | 32 (27-42) | 30 (26-38) | 29 (25-34) | 142.55 | 2 | <.001 |

*Note.* Tested with imputed dataset. Kruskal-Wallis chi-squared performed with log-transformed street trees density and richness

**Supplementary Table S5.** Log odds ratio of risk of antidepressant prescriptions by covariates, street tree density and street tree species richness. Continuous variables (tree density, richness, optimism and pessimism) were scaled to units of standard deviation.

|  | Log OR | SE | *p*-value | Lower CI | Upper CI |
| --- | --- | --- | --- | --- | --- |
| *Body Mass Index^1^* |  |  |  |  |  |
| Underweight | 0.13 | 0.55 | .817 | -0.95 | 1.20 |
| Overweight | 0.40 | 0.11 | <.001 | 0.19 | 0.62 |
| Obese | 0.42 | 0.12 | <.001 | 0.19 | 0.65 |
| *Age^2^* |  |  |  |  |  |
| Young (18-39) | -0.31 | 0.25 | .206 | -0.80 | 0.17 |
| Old age (65-79) | -0.51 | 0.11 | <.001 | -0.73 | -0.28 |
| *Gender^3^*  Female | 0.75 | 0.09 | <.001 | 0.57 | 0.93 |
| *Employment status^4^*  Employed | -0.73 | 0.11 | <.001 | -0.95 | -0.51 |
| *SES^5^* |  |  |  |  |  |
| Low | 0.15 | 0.16 | .346 | -0.16 | 0.45 |
| Medium | 0.10 | 0.14 | .456 | -0.17 | 0.37 |
| *Smoking^6^*  Current smoker | 0.22 | 0.10 | .033 | 0.02 | 0.43 |
| *Season^7^* |  |  |  |  |  |
| Spring | 0.27 | 0.12 | 0.02 | 0.03 | 0.50 |
| Autumn | -0.06 | 0.13 | 0.64 | -0.31 | 0.19 |
| Winter | 0.25 | 0.12 | .039 | 0.01 | 0.49 |
| Optimism^8^ | -0.41 | 0.04 | <.001 | -0.49 | -0.33 |
| Pessimism^8^ | 0.26 | 0.04 | <.001 | 0.17 | 0.34 |
| Street tree density, 100*m* | -0.09 | 0.05 | .057 | -0.18 | 0.00 |
| Street tree species richness, 100*m* | 0.03 | 0.06 | .588 | -0.09 | 0.15 |

*Note.* Tested with imputed dataset (*n* = 9751). OR = Odds Ratio. ^1^ = Reference group is Normal weight. ^2^ = Reference group is middle-aged (40-64). ^3^ = Reference group is Male. ^4^ = Reference group is unemployed. ^5^= Reference group is high SES. ^6^ = Reference group is current non-smoker. ^7^ = Reference group is summer. ^8^ = Continuous variable (range 0 = 12).

**Supplementary Table S6**. Stratified analyses assessing the effect of street tree density (scaled to units of standard deviation) at 100m around the home on antidepressant prescriptions for different levels of socio-economic status, gender and employment status.

|  | Antidepressants prescriptions | | |
| --- | --- | --- | --- |
|  | Log OR | SE | *p*-value |
| *SES* |  |  |  |
| Low SES x Street tree density 100m | -0.21 | 0.08 | .01 |
| Middle SES x Street tree density 100m | -0.02 | 0.06 | .72 |
| High SES x Street tree density 100m | -0.10 | 0.12 | .44 |
| *Gender* |  |  |  |
| Male x Street tree density 100m | -0.11 | 0.08 | .16 |
| Female x Street tree density 100m | -0.08 | 0.06 | .16 |
| *Employment status* |  |  |  |
| Unemployed x Street tree density 100m | -0.07 | 0.06 | .24 |
| Employed x Street tree density 100m | -0.13 | 0.08 | .09 |

*Note.* Tested with imputed dataset (*n* = 9751). OR = Odds Ratio

**3. Supplementary Figures**

**
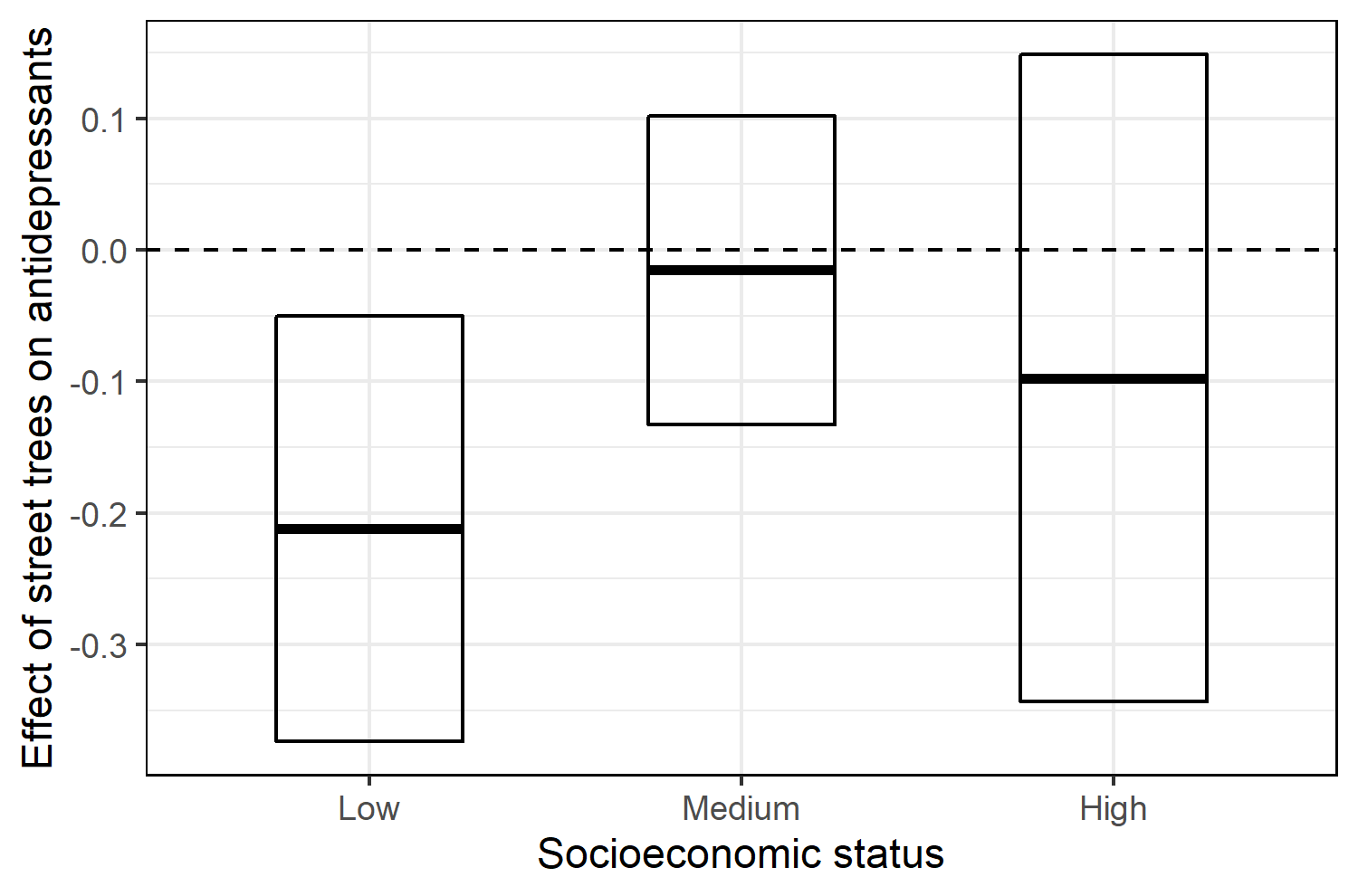
**

**Fig. S1.** Effect size of street tree density 100m on anti-depressants prescriptions for each SES group. Effect size (estimates 95% confidence intervals) represents the slopes of the relationship between log tree density at 100m and antidepressant prescriptions. Imputed dataset.

**4. Complete cases (original dataset) analyses**

**Supplementary Table S7.** Characteristics of the complete case (original) sample (*n* = 8453) and those who were (*n* = 488) and were not prescribed (*n* = 7965) antidepressants by socio-demographic, lifestyle and personality factors.

|  | Participants prescribed  antidepressants  (*n* = 488) | | Participants not prescribed antidepressants  (*n* = 7965) | | Total sample  (*n*=8453) |  |
| --- | --- | --- | --- | --- | --- | --- |
|  | *N* | % | *n* | % | *n* | % |
| *Age* |  |  |  |  |  |  |
| Mean (SD) | 57.79 (10.65) | | 56.52 (12.31) | | 56.60 (12.22) | |
| *Age group* |  |  |  |  |  |  |
| Young (18-39 years) | 10 | 2.0 | 432 | 5.5 | 442 | 5.0 |
| Middle (40-64 years) | 345 | 69.0 | 5199 | 65.2 | 5544 | 66.0 |
| Old age (65-79 years) | 133 | 26.6 | 2334 | 29.3 | 2467 | 30.0 |
| *Gender* |  |  |  |  |  |  |
| Male | 155 | 31.8 | 3896 | 48.9 | 4051 | 47.9 |
| Female | 333 | 68.2 | 4069 | 51.1 | 4402 | 52.1 |
| *Marital Status* |  |  |  |  |  |  |
| Married (living together or separately) | 285 | 58.4 | 4977 | 62.5 | 5262 | 62.3 |
| Never married | 76 | 15.6 | 1470 | 18.5 | 1546 | 18.3 |
| Divorced | 90 | 18.4 | 1090 | 13.7 | 1180 | 14.0 |
| Widowed | 37 | 7.6 | 428 | 5.4 | 465 | 5.5 |
| *Employment status* |  |  |  |  |  |  |
| Unemployed, or working less than 15 hrs/wk | 290 | 59.5 | 3411 | 42.7 | 3692 | 43.7 |
| Employed, or working 15+ hrs/wk | 198 | 40.6 | 4570 | 57.3 | 4761 | 56.3 |
| *Net Income (*€*)* |  |  |  |  |  |  |
| <1100 | 170 | 34.8 | 1632 | 20.5 | 1802 | 21.3 |
| 1100-2000 | 243 | 49.8 | 4389 | 55.1 | 4632 | 54.8 |
| >2000 | 75 | 15.4 | 1944 | 24.4 | 2019 | 23.9 |
| *Socio-economic status* |  |  |  |  |  |  |
| Low | 150 | 30.7 | 1419 | 17.8 | 1572 | 18.6 |
| Medium | 276 | 56.6 | 4825 | 60.6 | 5113 | 60.4 |
| High | 62 | 12.7 | 1721 | 21.6 | 1785 | 21.1 |
| *Body Mass Index* |  |  |  |  |  |  |
| Underweight | 3 | 0.6 | 43 | 0.5 | 47 | 0.6 |
| Normal weight | 123 | 25.2 | 2846 | 35.7 | 2976 | 35.1 |
| Overweight | 209 | 42.8 | 3183 | 40.0 | 3400 | 40.1 |
| Obese | 153 | 31.4 | 1893 | 23.8 | 2047 | 24.2 |
| *Smoking status* |  |  |  |  |  |  |
| Current non-smoker | 357 | 73.2 | 6220 | 78.1 | 6577 | 77.8 |
| Current smoker | 131 | 26.8 | 1745 | 21.9 | 1876 | 22.2 |
| *Alcohol consumption (g/day)* |  |  |  |  |  |  |
| <20g/day | 420 | 86.1 | 6341 | 79.6 | 6761 | 80.0 |
| ≥20g/day | 68 | 13.9 | 1624 | 20.4 | 1692 | 20.0 |
| *Season* |  |  |  |  |  |  |
| Winter | 119 | 21.1 | 1719 | 21.6 | 1838 | 21.7 |
| Spring | 145 | 29.7 | 1996 | 25.1 | 2141 | 25.3 |
| Summer | 121 | 24.8 | 2166 | 27.2 | 2287 | 27.1 |
| Autumn | 103 | 21.1 | 2084 | 26.2 | 2187 | 25.9 |
| Optimism^1^ |  |  |  |  |  |  |
| Mean (SD) | 7.48 (2.73) | | 8.90 (2.38) | | 8.81 (2.43) | |
| Pessimism^1^ |  |  |  |  |  |  |
| Mean (SD) | 5.43 (2.58) | | 4.41 (2.33) | | 4.47 (2.36) | |

*Note*. Complete cases (original) dataset (*n* = 8453). ^1^ = Continuous variable (range 0 -12).

**Supplementary Table S8.** Log odds ratio of risk of antidepressant prescriptions by covariates, street tree density and street tree species richness. Continuous variables (tree density, richness, optimism and pessimism) were scaled to units of standard deviation. Complete cases dataset (*n* = 8453).

|  | Log OR | SE | *p*-value | Lower CI | Upper CI |
| --- | --- | --- | --- | --- | --- |
| *Body Mass Index^1^* |  |  |  |  |  |
| Underweight | 0.19 | 0.64 | .766 | -1.06 | 1.44 |
| Overweight | 0.44 | 0.12 | <.001 | 0.21 | 0.68 |
| Obese | 0.46 | 0.13 | <.001 | 0.20 | 0.71 |
| *Age^2^* |  |  |  |  |  |
| Young (18-39) | -0.83 | 0.33 | .012 | -1.48 | -0.18 |
| Old age (65-79) | -0.62 | 0.13 | <.001 | -0.88 | -0.37 |
| *Gender^3^*  Female | 0.78 | 0.10 | <.001 | 0.58 | 0.98 |
| *Employment status^4^*  Employed | -0.74 | 0.12 | <.001 | -0.97 | -0.50 |
| *SES^5^* |  |  |  |  |  |
| Low | 0.22 | 0.17 | .200 | -0.12 | 0.56 |
| Medium | 0.11 | 0.15 | .462 | -0.18 | 0.40 |
| *Smoking^6^*  Current smoker | 0.17 | 0.11 | .136 | -0.05 | 0.39 |
| *Season*^7^  Spring | 0.21 | 0.13 | .116 | -0.05 | 0.46 |
| Autumn | -0.11 | 0.14 | .427 | -0.39 | 0.16 |
| Winter | 0.18 | 0.14 | .186 | -0.09 | 0.45 |
| Optimism^8^ | -0.43 | 0.05 | <.001 | -0.52 | -0.34 |
| Pessimism^8^ | 0.25 | 0.05 | <.001 | 0.15 | 0.34 |
| Street tree density, 100*m* | -0.11 | 0.05 | .039 | -0.21 | -0.01 |
| Street tree species richness, 100*m* | 0.02 | 0.07 | .771 | -0.11 | 0.15 |

*Note.* Complete cases (original) dataset (*n* = 8453). OR = Odds Ratio. ^1^ = Reference group is Normal weight. ^2^ = Reference group is middle-aged (40-64). ^3^ = Reference group is Male. ^4^ = Reference group is unemployed. ^5^= Reference group is high SES. ^6^ = Reference group is current non-smoker. ^7^ = Reference group is summer. ^8^ = Continuous variable (range 0 = 12).


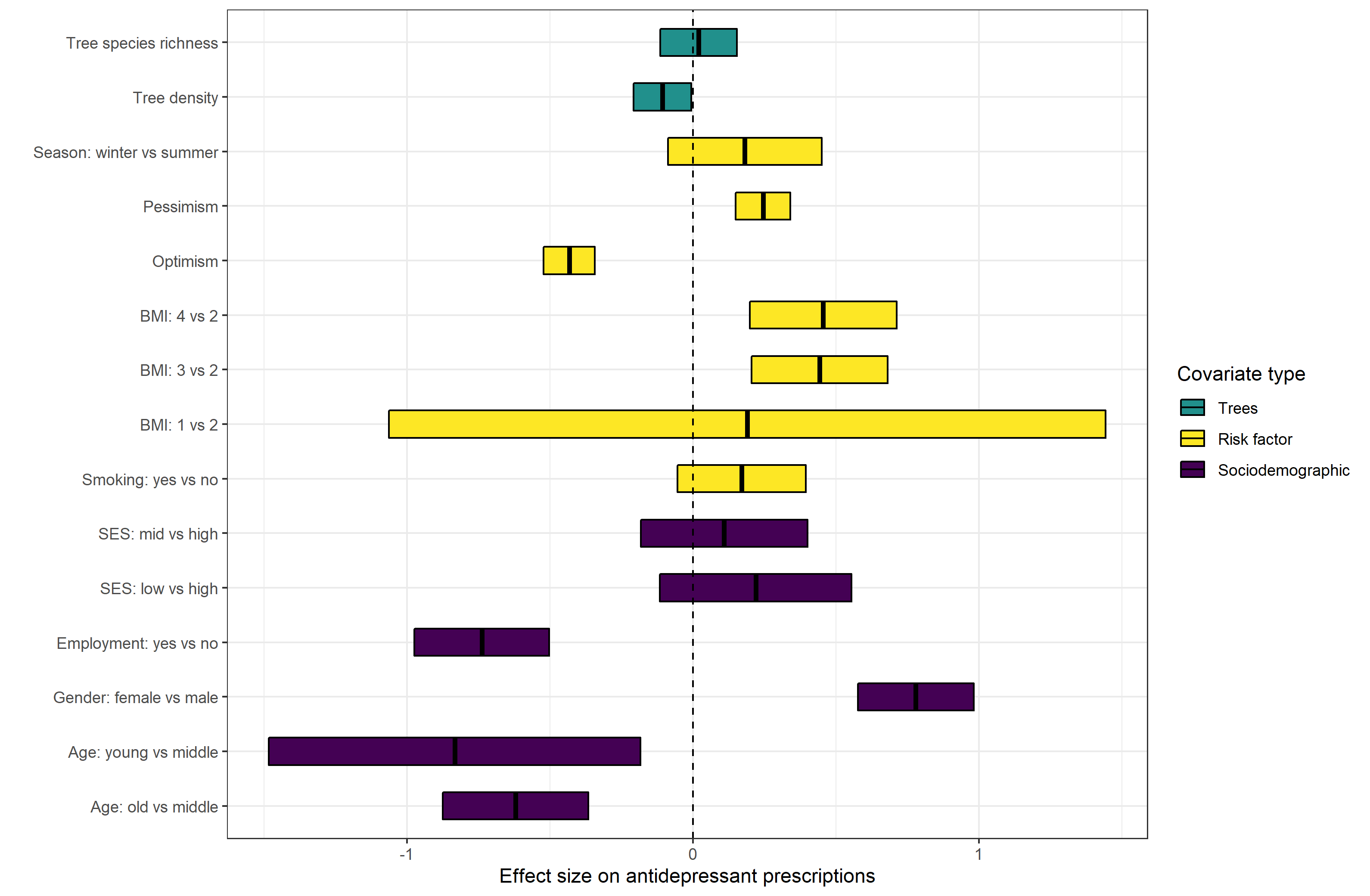


**Fig. S2. Effect size of covariates and street tree density and richness at 100m on antidepressant prescriptions. Shown are the regression coefficients (change in log OR) and 95% confidence intervals. Regression coefficients for continuous variables (pessimism, optimism, tree richness and density) were scaled to units of standard deviation; for the categorical variables, the effect sizes represent differences between levels. The dashed line is the line of no effect. BMI (Body Mass Index): 1=underweight, 2=normal weight, 3=overweight, 4=obese**. **Age: young=18-39 years; middle=40-64 years; old=65-79 years. Complete cases dataset (*n* = 8453).**

**
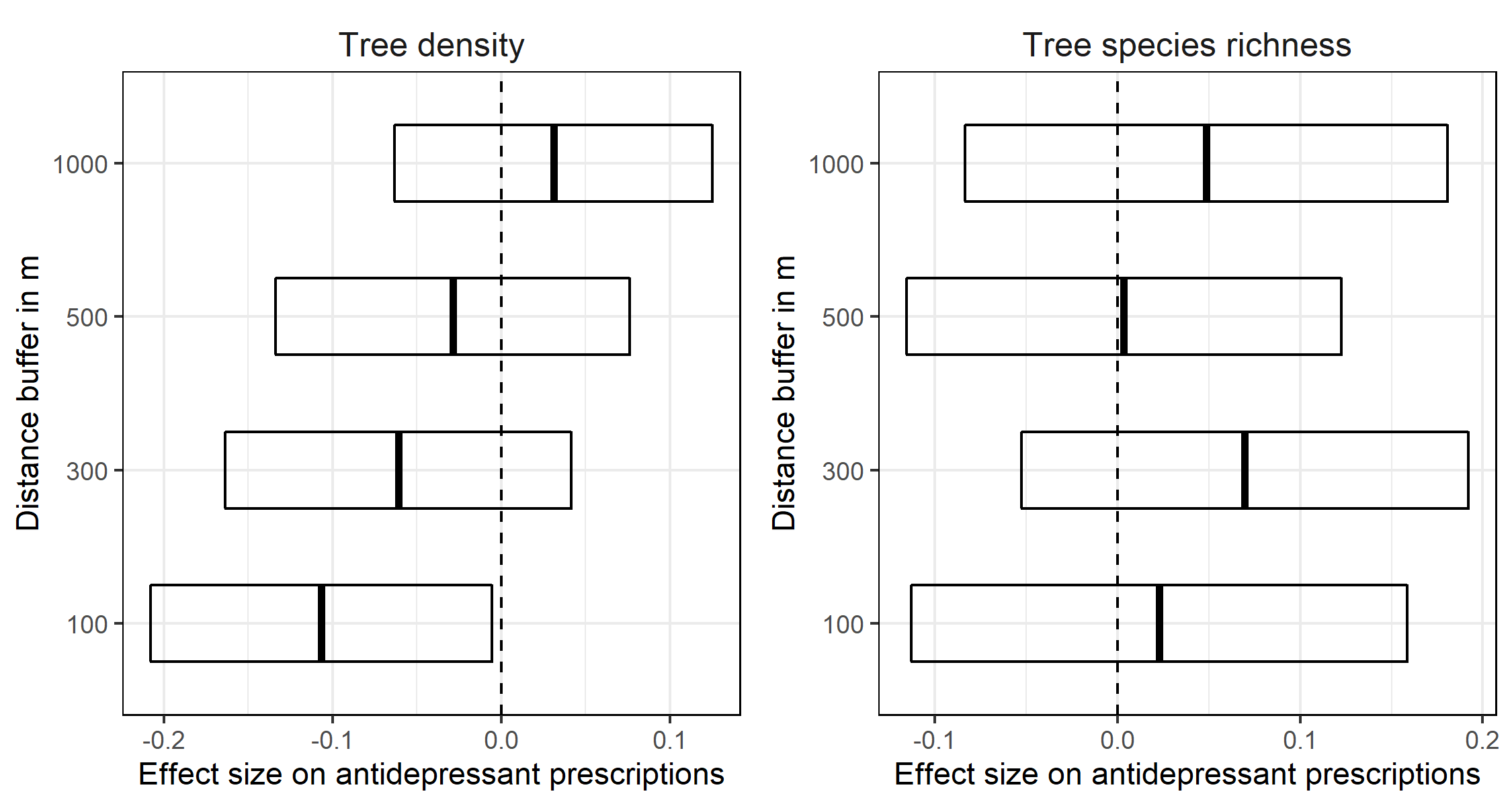
**

**Fig. S3. Effect size of density and species richness of street trees at different spatial proximities around the participants’ home (buffer widths) on antidepressant prescriptions. Street tee density and richness were standardized to units of standard deviation prior to analysis. Complete cases dataset.**

**Supplementary Table S9**. Moderation results assessing the interaction of street tree density (scaled to units of standard deviation) at 100m around the home and socio-economic status (SES), gender and employment status on antidepressant prescriptions.

|  | Antidepressants prescriptions | | |
| --- | --- | --- | --- |
|  | Log OR | SE | *p*-value |
| *SES* |  |  |  |
| Low SES x Street tree density 100m | -0.26 | 0.09 | .01 |
| Middle SES x Street tree density 100m | -0.04 | 0.07 | .51 |
| High SES x Street tree density 100m | -0.05 | 0.13 | .73 |
| *Gender* |  |  |  |
| Male x Street tree density 100m | -0.11 | 0.09 | .22 |
| Female x Street tree density 100m | -0.11 | 0.06 | .09 |
| *Employment status* |  |  |  |
| Unemployed x Street tree density 100m | -0.09 | 0.07 | .18 |
| Employed x Street tree density 100m | -0.14 | 0.08 | .09 |

*Note.* Complete case (original) dataset (n = 8453). OR = Odds Ratio


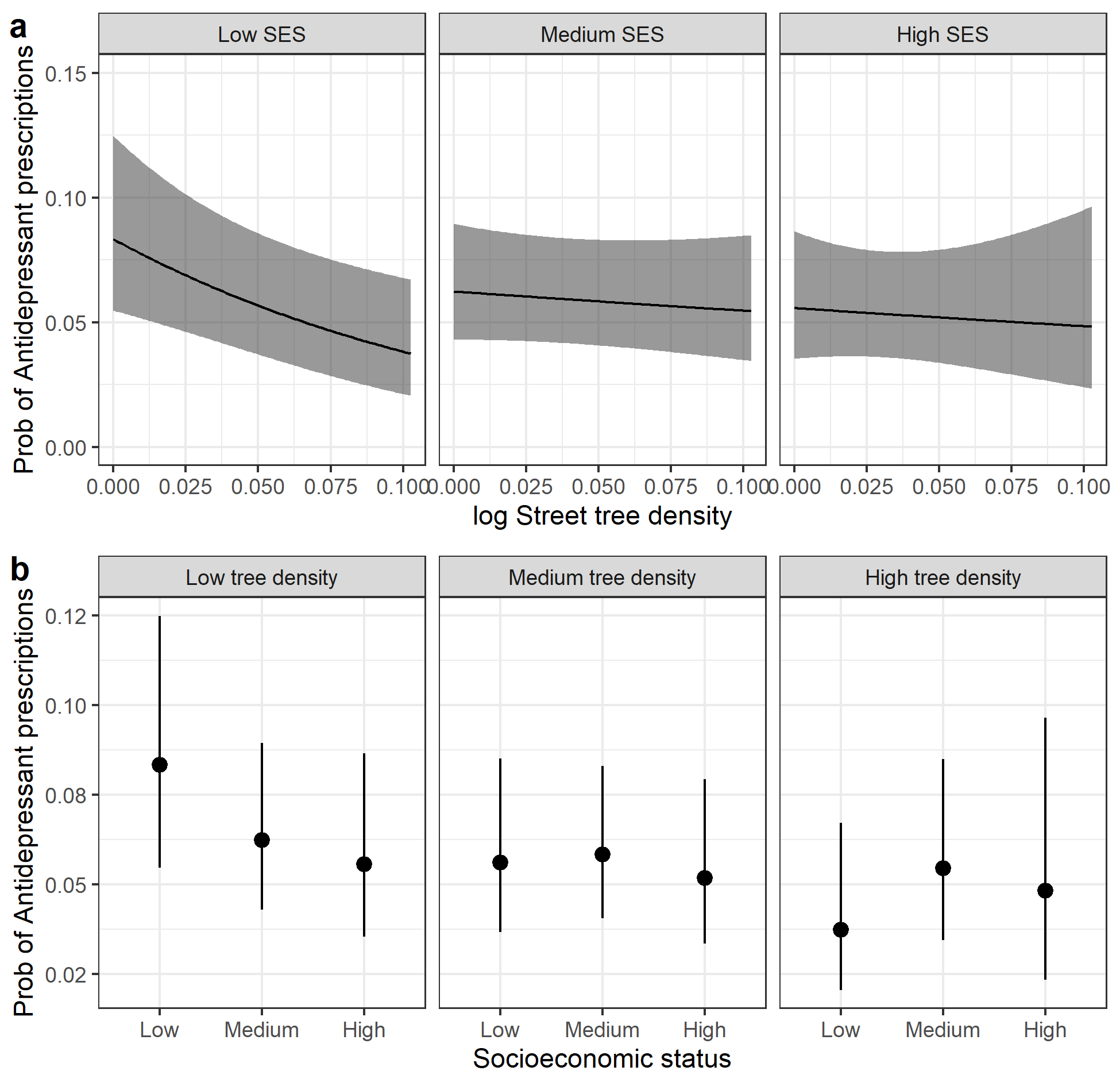


**Fig. S4. Modelled probability of antidepressant prescriptions as a function of street tree density 100m around the home and individual socio-economic status (SES). The black line is the mean and the shade area are the 95% confidence intervals. The regression is statistically significant (P<0.01) for low SES individuals for not for medium or high SES. Complete cases dataset.**

**5. Supplementary References**

1. Boyle, B. *et al.* The taxonomic name resolution service: an online tool for automated standardization of plant names. *BMC Bioinformatics* **14**, 16 (2013).

2. Taxonomic Name Resolution Service. iPlant Collaborative. Version 4.0. http://tnrs.iplantcollaborative.org/TNRSapp.html (2018).

3. Loeffler, M. *et al.* The LIFE-Adult-Study: Objectives and design of a population-based cohort study with 10,000 deeply phenotyped adults in Germany. *BMC Public Health* **15**, 691 (2015).

4. WHO Collborating Centre for Drug Statistics Methodology. ATC/DDD Index: N06A Antidepressants. https://www.whocc.no/atc_ddd_index/?code=N06A (2019).

5. Geofabrik. Download OpenStreetMap data for Sachen. http://download.geofabrik.de/europe/germany/sachsen.html (2019).

6. Cipeluch, B., Jacob, R., Winstanley, A. & Mooney, P. Comparison of the accuracy of OpenStreetMap for Ireland with Google Maps and Bing Maps. in *Proceedings of the Ninth International Symposium on Spatial Accuracy Assessment in Natural Resources and Enviromental Sciences* 337 (2010).

7. Helbich, M., Amelunxen, C. & Neis, P. Comparative Spatial Analysis of Positional Accuracy of OpenStreetMap and Proprietary Geodata. *Proc. GI_Forum 2012 Geovisualization, Soc. Learn.* 24–33 (2012).

8. Zhang, H. & Malczewski, J. Accuracy Evaluation of the Canadian OpenStreetMap Road Networks. *Int. J. Geospatial Environ. Res.* **5**, (2018).

9. El-Ashmawy, K. L. A. Testing the positional accuracy of OpenStreetMap data for mapping applications. *Geod. Cartogr.* **42**, 25–30 (2016).

10. Lampert, T., Kroll, L., Müters, S. & Stolzenberg, H. Measurement of socioeconomic status in the German health interview and examination survey for adults (DEGS1). *Bundesgesundheitsblatt - Gesundheitsforsch. - Gesundheitsschutz* **56**, 631–636 (2013).

11. Fan, Y., Das, K. V & Chen, Q. Neighborhood green, social support, physical activity, and stress: Assessing the cumulative impact. *Health Place* **17**, 1202–1211 (2011).

12. Hinz, A. *et al.* Psychometric properties of the Satisfaction with Life Scale (SWLS), derived from a large German community sample. *Qual. Life Res.* **27**, 1661–1670 (2018).

13. Hinz, A. *et al.* Psychometric evaluation of the Generalized Anxiety Disorder Screener GAD-7, based on a large German general population sample. *J. Affect. Disord.* **210**, 338–344 (2017).

14. Astell-Burt, T., Mitchell, R. & Hartig, T. The association between green space and mental health varies across the lifecourse. a longitudinal study. *J. Epidemiol. Community Health* (2014) doi:10.1136/jech-2013-203767.

15. World Health Organization. Body mass index - BMI. http://www.euro.who.int/en/health-topics/disease-prevention/nutrition/a-healthy-lifestyle/body-mass-index-bmi (2018).

16. Glaesmer, H. *et al.* Psychometric properties and population-based norms of the Life Orientation Test Revised (LOT-R). *Br. J. Health Psychol.* **17**, 432–445 (2012).

17. Herzberg, P. Y., Glaesmer, H. & Hoyer, J. Separating optimism and pessimism: A robust psychometric analysis of the revised Life Orientation Test (LOT-R). *Psychol. Assess.* **18**, 433–438 (2006).

18. Scheier, M. F., Carver, C. S. & Bridges, M. W. Distinguishing optimism from neuroticism (and trait anxiety, self-mastery, and self-esteem): A re-evaluation of the Life Orientation Test. *J. Pers. Soc. Psychol.* **67**, 1063–1078 (1994).
